# Reciprocal associations among fear of failure, academic buoyancy, and academic procrastination in primary school students: a three-wave cross-lagged panel study

**DOI:** 10.3389/fpsyg.2026.1847679

**Published:** 2026-10-01

**Authors:** Jiayuan Fang, Nan Jiang, Yifan Wei, Yanzhen Zhang

**Affiliations:** 1 School of Education, Jiangxi Normal University, Nanchang, China; 2 Mental Health Education and Counseling Center, Jiangxi Arts & Ceramics Technology Institute, Jingdezhen, China; 3 Zhuhai Convention & Exhibition Group Co., Ltd., Zhuhai, China; 4 Jingdezhen Ceramic University, Jingdezhen, China; 5 Department of Statistics, Donald Bren School of Information and Computer Sciences, University of California, Irvine, Irvine, CA, United States

**Keywords:** academic buoyancy, academic procrastination, elementary school students, fear of failure, longitudinal study

## Abstract

Academic procrastination is common in late elementary school and is associated with lower academic achievement and poorer well-being. Drawing on achievement motivation theory, the Study Demands-Resources (SD-R) framework, academic buoyancy theory, and Conservation of Resources theory, this three-wave longitudinal study examined reciprocal associations among fear of failure, academic buoyancy, and academic procrastination in Grade 5–6 students (*N* = 542). Participants completed self-report measures of fear of failure, academic buoyancy, and academic procrastination at three time points across one academic year, with approximately 3 months between adjacent waves. Covariate-adjusted cross-lagged panel models were estimated to examine reciprocal prospective associations among the three constructs. In the covariate-adjusted traditional CLPM, fear of failure and academic buoyancy prospectively predicted each other in negative directions, and academic buoyancy and academic procrastination also showed negative reciprocal prospective associations. Fear of failure predicted later procrastination from T1 to T2, whereas the direct T2-to-T3 path was not significant. Academic buoyancy showed significant longitudinal indirect associations linking fear of failure with later procrastination and procrastination with later fear of failure. A supplementary RI-CLPM indicated that the within-person cross-lagged effects were not uniform across intervals and that the within-person indirect effects were not statistically significant. These findings suggest that academic buoyancy may function as a modifiable adaptive coping capacity that helps students cope with fear of failure and is associated with lower subsequent academic procrastination during the transition from late primary school to middle school.

## 1 Introduction

Academic procrastination is commonly described as a failure of self-regulation in which students voluntarily delay intended academic tasks despite anticipating that such delay may produce negative consequences (Steel, 2007). Procrastination is particularly consequential in competitive educational settings, where rising performance demands may compromise students’ academic adjustment. During the transition from primary to middle school, students encounter increasing homework loads, evaluation frequency, and expectations for independent learning; these conditions may intensify the need for self-regulation (Yue et al., 2024). Persistent procrastination has been associated with lower achievement and greater distress, including test anxiety, self-blame, reduced self-worth, and helplessness (Wang et al., 2022). As blended and digital learning environments become more common, younger students may also face increased distraction and higher demands for self-management (King et al., 2022). Identifying modifiable psychological processes underlying procrastination is therefore important for promoting academic well-being in elementary school students.

Previous studies have linked academic procrastination to personality factors, task characteristics, and contextual influences. However, less is known about how achievement-related threat appraisals and adaptive academic coping capacities are prospectively related during late primary school. Fear of failure is a central negative achievement-related emotion and motivational tendency, commonly understood as an avoidance-oriented motive in evaluative settings (Atkinson, 1957; Elliot and Church, 1997). Students high in fear of failure tend to anticipate shame, self-worth threat, or negative social evaluation when they fail (Conroy, 2001). Such appraisals may make academic tasks feel psychologically threatening and may increase avoidance-based strategies, including procrastination (Haghbin et al., 2012). Nevertheless, fear of failure does not inevitably translate into procrastination. This suggests that adaptive coping capacities may shape whether achievement threat develops into maladaptive delay behavior (Narciss and Alemdag, 2025).

Academic buoyancy may represent one such capacity. Academic buoyancy refers to students’ ability to deal with everyday academic setbacks, challenges, and pressures, such as low quiz scores, heavy homework, or routine classroom difficulties (Martin and Marsh, 2008; Martin, 2013). It should be distinguished from academic resilience, which typically refers to adaptive functioning under severe, chronic, or major adversity. In this sense, academic buoyancy is not a general resilience trait or a static stock of psychological resources; rather, it reflects a day-to-day adaptive academic coping capacity that enables students to maintain engagement and recover from minor academic adversity (Lakkavaara et al., 2024).

The present study integrates four complementary perspectives. Achievement motivation theory helps explain fear of failure as an avoidance-oriented motive rooted in anticipated shame, loss of competence, and self-worth threat (Atkinson, 1957; Elliot and Church, 1997). The Study Demands-Resources (SD-R) framework suggests that academic demands may strain students, whereas personal and contextual resources can support engagement and protect against maladaptive outcomes (Lesener et al., 2020; Salmela-Aro and Upadyaya, 2014). Academic buoyancy theory clarifies the specific coping capacity relevant to everyday school setbacks (Martin and Marsh, 2008). Finally, Conservation of Resources (COR) theory offers a broader framework for understanding how perceived resource threat and resource protection processes may unfold over time (Hobfoll, 1989). Together, these perspectives suggest that fear of failure, academic buoyancy, and academic procrastination may operate as a dynamic system rather than as isolated constructs.

### 1.1 Fear of failure and academic buoyancy in achievement and study demands-resources (SD-R) contexts

Fear of failure is commonly defined as a tendency to avoid failure because of anticipated negative consequences in achievement contexts, including shame, social devaluation, and perceived loss of self-worth (Conroy, 2001; Taylor et al., 2023). From the perspective of achievement motivation theory, fear of failure represents a motivational vulnerability that makes evaluative academic situations more threatening. When students interpret academic tasks as tests of personal worth, cognitive and emotional resources may be redirected toward threat monitoring, self-protection, and rumination rather than toward task-focused coping (Dang and Middlemiss, 2025).

Academic buoyancy, by contrast, refers to students’ capacity to respond adaptively to ordinary academic setbacks and pressures (Martin and Marsh, 2008; Martin, 2013). It is conceptually related to self-efficacy, perceived control, persistence, and low anxiety, but it is not identical to any of these constructs. Its defining feature is the capacity to remain engaged and recover from everyday academic difficulties. Recent work has similarly emphasized academic buoyancy as a proximal academic coping capacity that helps students manage test anxiety, stress, setbacks, and maladaptive coping patterns (Putwain et al., 2023; Putwain et al., 2024).

Fear of failure and academic buoyancy may therefore be negatively related over time. High fear of failure may undermine subsequent buoyancy by increasing threat appraisal, rumination, and emotional burden in routine academic situations (Balkis et al., 2024). Students who are preoccupied with possible failure may find it more difficult to appraise setbacks as manageable and to mobilize adaptive coping strategies. Conversely, higher academic buoyancy may reduce subsequent fear of failure because buoyant students are more likely to appraise ordinary setbacks as temporary, specific, and improvable. Prior longitudinal work suggests that buoyant students tend to show higher perceived control and more adaptive reappraisal, which may reduce the emotional salience of possible failure (Martin et al., 2010). In this sense, academic buoyancy is better understood as an adaptive coping capacity that helps students appraise everyday academic setbacks as manageable challenges rather than threats, instead of as a passive reservoir that is simply consumed by fear.

### 1.2 Reciprocal links between academic buoyancy and academic procrastination

Academic procrastination is generally viewed as a self-regulatory breakdown in which individuals delay academic tasks despite knowing that delay may be harmful (Steel, 2007). According to Temporal Motivation Theory, procrastination reflects the joint influence of task value, expectancy, impulsiveness, and delay, with short-term relief often outweighing long-term costs (Gonzalez-Brignardello et al., 2023). Consistent with this account, recent research links procrastination to negative affect and emotion-regulation difficulties, suggesting that procrastination can function as an emotional avoidance strategy (Sirois and Pychyl, 2013; Netzer Turgeman and Pollak, 2023; Nie et al., 2025).

From an adaptive coping perspective, academic buoyancy may reduce later procrastination because buoyant students are more able to remain engaged when academic tasks become difficult, frustrating, or evaluative. Rather than disengaging to obtain immediate emotional relief, they may use problem-focused coping, cognitive reappraisal, and persistence. Previous studies indicate that academic buoyancy is related to less avoidant or dysfunctional coping and to more adaptive academic outcomes (Nordby et al., 2017; Putwain et al., 2024; Shi et al., 2024).

The reverse association is also plausible. Procrastination may erode later academic buoyancy by increasing time pressure, reducing task mastery experiences, and accumulating unfinished work or perceived failure (Yosopov et al., 2024). These consequences may weaken students’ sense that they can manage ordinary academic pressures. Repeated delay may therefore make future setbacks feel less controllable and more threatening. Longitudinal research on procrastination and emotion regulation similarly suggests that maladaptive delay and negative emotional processes can reinforce each other over time (Chen and Chung, 2025).

### 1.3 An integrated longitudinal model and indirect-association hypothesis

Taken together, fear of failure, academic buoyancy, and academic procrastination may form an interdependent longitudinal system. Fear of failure may prospectively predict higher procrastination because achievement threat can increase avoidance-oriented self-regulation. However, the association between fear of failure and later procrastination may also operate through reduced academic buoyancy. From an integrated demands-resources and COR perspective, fear of failure can be regarded as a perceived achievement-related threat that interferes with the mobilization of adaptive coping capacities. Lower academic buoyancy may then make students more likely to rely on avoidance-based procrastination when facing difficult or evaluative academic tasks.

A reverse pathway may also occur. Procrastination may predict higher later fear of failure by contributing to accumulated time pressure, incomplete assignments, and poorer performance experiences. These outcomes may heighten students’ expectations of failure and weaken their confidence in managing ordinary academic setbacks, thereby reducing academic buoyancy and increasing later fear of failure. Thus, academic buoyancy may serve as a longitudinal mechanism linking both emotional threat and maladaptive delay behavior.

Because the present study used a complete reciprocal cross-lagged panel model, additional direct cross-lagged paths among the three constructs were estimated even when they were not focal hypotheses. These paths were included to provide a conservative test of the hypothesized relations by accounting for competing prospective associations among the constructs.

### 1.4 The present study

This three-wave longitudinal study examined reciprocal associations among fear of failure, academic buoyancy, and academic procrastination in upper elementary school students. Based on the integrated theoretical framework described above, we proposed the following hypotheses (Figure 1):

**Figure 1:**
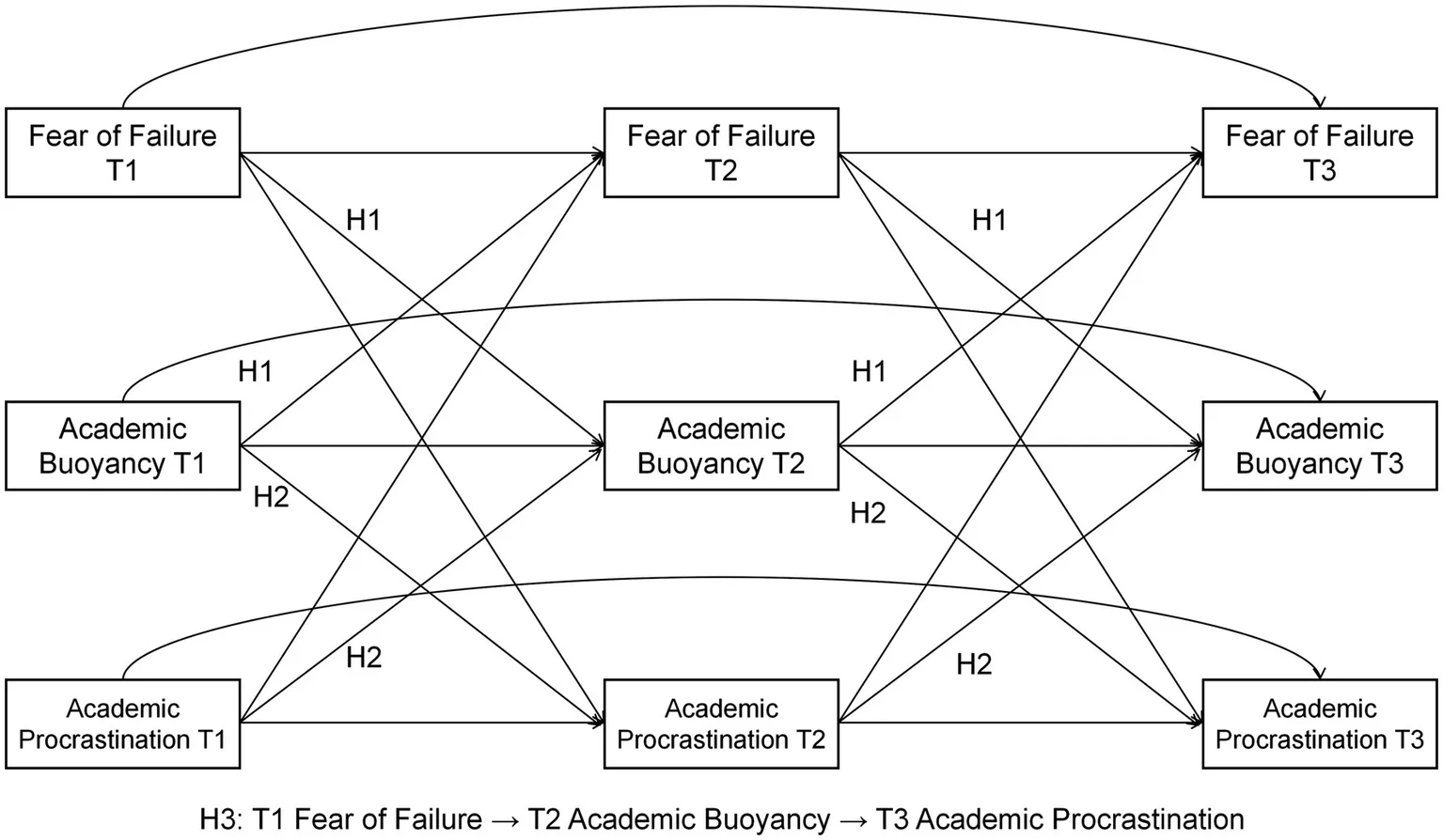
The hypothesized cross-lagged panel model. H1 and H2 indicate the focal reciprocal prospective associations. H3 indicates the longitudinal indirect pathway from T1 fear of failure to T3 academic procrastination through T2 academic buoyancy. The reverse indirect pathway from T1 academic procrastination to T3 fear of failure through T2 academic buoyancy was examined as an exploratory pathway. Additional direct cross-lagged paths were estimated as competing paths in the full reciprocal model.

*H1*: Fear of failure and academic buoyancy will reciprocally predict each other negatively over time.

*H2*: Academic buoyancy and academic procrastination will reciprocally predict each other negatively over time.

*H3*: Academic buoyancy will show longitudinal indirect associations linking fear of failure with later academic procrastination. The reverse indirect pathway from academic procrastination to later fear of failure through academic buoyancy was examined as an exploratory pathway.

## 2 Methods

### 2.1 Participants

A total of 598 fifth- and sixth-grade students were recruited from one primary school in Jiangxi Province, China. The school was selected through convenience sampling. Fourth-grade students were not included because class reorganization at that grade level made it infeasible for teachers to administer three waves of measurement as scheduled. Participant attrition across the three waves occurred because of school transfer, absence, and invalid or incomplete questionnaires. The final analytic sample comprised 542 students with usable longitudinal data. The sample included 272 girls and 270 boys, 410 fifth-grade students and 132 sixth-grade students, and 38 only children and 504 non-only children. The mean age was 10.74 years (SD = 0.78).

Little’s missing completely at random (MCAR) test was significant, chi-square (225) = 289.39, *p* < 0.01, indicating that the missingness mechanism was not completely at random (Little, 1988). Full information maximum likelihood (FIML) estimation was therefore used to handle missing data in model estimation. FIML has been shown to perform favorably compared with alternative missing-data approaches and may yield less biased parameter estimates even when missingness deviates from complete randomness (Muthen et al., 1987). In addition, independent-samples t tests comparing baseline scores between participants who completed all waves and those who dropped out showed no significant differences in fear of failure (*t* = −1.76, *p* > 0.05), academic buoyancy (*t* = 1.50, *p* > 0.05), or academic procrastination (*t* = −1.89, *p* > 0.05), suggesting no evidence of systematic attrition on the focal variables.

### 2.2 Measures

For all measures, scale scores were computed as total-score composites and used as observed indicators in the structural models. Internal consistency was evaluated using both Cronbach’s alpha and McDonald’s omega. Accordingly, the omega coefficients reported below refer to the reliability of the corresponding total-score composites rather than to separate subscale-level reliability estimates.

Fear of failure. Fear of failure was assessed using the short form of the Performance Failure Appraisal Inventory (PFAI; Conroy, 2001). The scale assesses students’ concerns about negative consequences of failure, including shame, embarrassment, and devaluation of self-worth. Students responded to five items (e.g., “When I am not succeeding, I get down on myself”) on a 5-point Likert scale ranging from 1 (strongly disagree) to 5 (strongly agree). Higher scores indicated greater fear of failure. Cronbach’s alpha coefficients were 0.83, 0.89, and 0.91 from T1 to T3, and McDonald’s omega coefficients were 0.83, 0.89, and 0.91, respectively.

Academic buoyancy. Academic buoyancy was measured using a 4-item Academic Buoyancy Scale adapted for Chinese students (Sun, 2009). The scale reflects students’ perceived capacity to cope with ordinary school-related academic setbacks and pressures (e.g., “I think I am good at dealing with the pressures of schoolwork”). Students responded on a 5-point Likert scale ranging from 1 (strongly disagree) to 5 (strongly agree). Higher scores indicated greater academic buoyancy. Cronbach’s alpha coefficients were 0.72, 0.77, and 0.83 from T1 to T3, and McDonald’s omega coefficients were 0.72, 0.78, and 0.83, respectively. Because the present study used an adapted 4-item version, longitudinal measurement invariance was examined before testing structural relations.

Academic procrastination. Academic procrastination was assessed using the Academic Procrastination Questionnaire for Primary School Students (Wang, 2010). The questionnaire measures three dimensions: delay in academic behavior, lack of planning and execution, and negative academic attitudes. Students responded to 19 items (e.g., “I often wait until the last minute to start my homework”) on a 5-point Likert scale ranging from 1 (strongly disagree) to 5 (strongly agree). Higher scores indicated greater academic procrastination. Cronbach’s alpha coefficients were 0.93, 0.95, and 0.96 from T1 to T3, and McDonald’s omega coefficients were 0.93, 0.95, and 0.96, respectively. Although this questionnaire includes three dimensions, the present study used the total-score composite in the CLPM; therefore, the reported omega coefficients refer to the reliability of the total-score composite rather than to subscale-level reliability estimates. These results indicated acceptable to excellent internal consistency across waves.

### 2.3 Procedure

This study was approved by the Ethics Committee of the Mental Health Education and Counseling Center, Jiangxi Arts & Ceramics Technology Institute (approved on September 1, 2024). Written consent was obtained from the school, parents or guardians, and students before participation. Data were collected in classrooms at three time points across one academic year, with approximately 3 months between adjacent waves. School psychology teachers administered the questionnaires, which took approximately 20 min to complete.

### 2.4 Data analysis

Data were entered in EpiData and analyzed in SPSS 27.0 for preliminary analyses, including descriptive statistics, Pearson correlations, and independent-samples *t*-tests for demographic group differences. Common method bias was assessed at each wave by comparing measurement models with and without a common latent method factor (Richardson et al., 2009). Unconditional linear latent growth models were estimated to describe mean-level changes in fear of failure, academic buoyancy, and academic procrastination across the three waves.

All structural equation models were estimated in Mplus 8.3 using maximum likelihood estimation with full information maximum likelihood (FIML) to handle missing data. Responses outside the intended 1–5 range were treated as missing. Scale scores were computed when at least 80% of the corresponding items had valid responses; otherwise, the scale score for that wave was coded as missing. FIML allowed all available information to contribute to model estimation without imputing individual missing values.

Confirmatory factor analyses and nested model comparisons were used to examine longitudinal measurement invariance, including configural, metric, and scalar invariance. Cross-lagged panel models (CLPMs) were used to examine prospective reciprocal associations among the three constructs while accounting for autoregressive stability. For model comparison, scale scores were standardized within each wave before equality constraints were imposed across adjacent intervals. Gender (1 = boy, 2 = girl) and grade level (5 or 6) were included as time-invariant covariates predicting each construct across the three waves. Longitudinal mediation was tested with 5,000 bootstrap resamples; indirect effects were considered significant when the 95% bootstrap confidence interval did not include zero. As a robustness analysis, a random-intercept cross-lagged panel model (RI-CLPM) was estimated to examine whether the main findings were retained after separating stable between-person differences from within-person fluctuations.

## 3 Results

### 3.1 Common method bias

To evaluate potential common method bias, we conducted latent method factor analyses and compared measurement models with and without a common method factor at each wave. For Wave 1, the model without the common method factor showed acceptable fit (chi-square/df = 2.78, TLI = 0.90, CFI = 0.91, RMSEA = 0.06). Adding a common method factor did not improve model fit (chi-square/df = 3.17, TLI = 0.88, CFI = 0.89, RMSEA = 0.06). A similar pattern emerged across subsequent waves. For Wave 2, the model without the common method factor showed acceptable fit (chi-square/df = 2.84, TLI = 0.92, CFI = 0.93, RMSEA = 0.06), whereas the model with the method factor showed poorer fit (chi-square/df = 3.43, TLI = 0.89, CFI = 0.90, RMSEA = 0.07). For Wave 3, the model without the method factor (chi-square/df = 3.37, TLI = 0.91, CFI = 0.92, RMSEA = 0.07) also fit better than the model with the method factor (chi-square/df = 4.05, TLI = 0.89, CFI = 0.90, RMSEA = 0.08). Detailed comparisons are provided in Supplementary Table S5. Overall, common method bias did not appear to be a major threat to the findings.

### 3.2 Descriptive statistics and correlations

Descriptive statistics and correlations are summarized in Table 1. Across the three waves, fear of failure was positively correlated with academic procrastination and negatively correlated with academic buoyancy. Academic buoyancy was negatively correlated with academic procrastination. Cross-wave correlations were moderate to strong, indicating substantial temporal stability in the constructs.

**Table 1:** Descriptive statistics and correlations among study variables.

Variable	*M*	SD	1	2	3	4	5	6	7	8	9
1. T1 Fear of failure	2.49	1.08	–								
2. T2 Fear of failure	2.55	1.14	0.71***	–							
3. T3 Fear of failure	2.62	1.14	0.60***	0.72***	–						
4. T1 Academic buoyancy	3.76	0.96	−0.40***	−0.41***	−0.40***	–					
5. T2 Academic buoyancy	3.69	0.98	−0.51***	−0.58***	−0.53***	0.53***	–				
6. T3 Academic buoyancy	3.63	1.03	−0.45***	−0.56***	−0.55***	0.48***	0.61***	–			
7. T1 Academic procrastination	1.98	0.78	0.50***	0.53***	0.45***	−0.48***	−0.52***	−0.44***	–		
8. T2 Academic procrastination	2.01	0.81	0.48***	0.57***	0.48***	−0.44***	−0.54***	−0.49***	0.78***	–	
9. T3 Academic procrastination	2.10	0.86	0.44***	0.51***	0.54***	−0.37***	−0.53***	−0.49***	0.70***	0.76***	–

*N* = 542. 1 = T1 fear of failure; 2 = T2 fear of failure; 3 = T3 fear of failure; 4 = T1 academic buoyancy; 5 = T2 academic buoyancy; 6 = T3 academic buoyancy; 7 = T1 academic procrastination; 8 = T2 academic procrastination; 9 = T3 academic procrastination. *p < 0.05, **p < 0.01, ***p < 0.001.

### 3.3 Demographic differences in study variables

Independent-samples t tests were conducted to examine whether study variables differed by gender, grade, or only-child status. No statistically significant gender differences were found in academic procrastination at any wave. Girls reported higher fear of failure than boys at T1 and T2, whereas boys reported higher academic buoyancy than girls at T2. Grade-level differences were also observed: sixth-grade students reported higher fear of failure and academic procrastination and lower academic buoyancy than fifth-grade students. No significant differences were found between only children and students with siblings. Detailed results are presented in Supplementary Tables S1–S3.

### 3.4 Mean-level change across waves

Unconditional linear growth models were estimated to describe mean-level change across the three waves. On average, fear of failure increased over time (slope mean = 0.060, *p* = 0.005), and academic procrastination also increased (slope mean = 0.060, *p* < 0.001). In contrast, academic buoyancy decreased over time (slope mean = −0.072, *p* = 0.002). These trends suggest that late primary school may be characterized by increasing achievement-related threat and procrastination together with declining perceived capacity to manage everyday academic setbacks. Detailed model estimates are provided in Supplementary Table S4.

### 3.5 Longitudinal measurement invariance

Before testing the structural models, we examined whether the measurement structure was comparable across the three waves. Confirmatory factor analyses were used to test configural, metric, and scalar invariance. As shown in Table 2, changes in model fit were negligible and met recommended criteria (△CFI ≤ 0.01; △RMSEA ≤ 0.015). These results indicate that fear of failure, academic buoyancy, and academic procrastination were measured equivalently across time, supporting the validity of longitudinal comparisons.

**Table 2:** Longitudinal measurement invariance tests for fear of failure, academic buoyancy, and academic procrastination.

Variable	Model	chi-square	df	CFI	TLI	SRMR	RMSEA	Comparison	△CFI	△RMSEA
Fear of failure	M1	205.99	72	0.963	0.946	0.034	0.059			
	M2	218.94	80	0.961	0.949	0.036	0.057	M2-M1	−0.002	−0.002
	M3	254.41	88	0.954	0.945	0.037	0.059	M3-M2	−0.007	0.002
Academic buoyancy	M1	49.42	39	0.993	0.989	0.025	0.022			
	M2	60.12	45	0.990	0.986	0.034	0.025	M2-M1	−0.003	0.003
	M3	67.22	51	0.990	0.987	0.037	0.024	M3-M2	0.000	−0.001
Academic procrastination	M1	2696.55	1,446	0.913	0.904	0.042	0.040			
	M2	2758.42	1,478	0.911	0.904	0.045	0.040	M2-M1	−0.002	0.000
	M3	2845.74	1,510	0.907	0.902	0.045	0.040	M3-M2	−0.004	0.000

M1 = configural invariance model; M2 = metric invariance model; M3 = scalar invariance model. CFI = Comparative Fit Index; TLI = Tucker–Lewis Index; SRMR = Standardized Root Mean Square Residual; RMSEA = Root Mean Square Error of Approximation.

### 3.6 Cross-lagged panel models

Four cross-lagged panel models were compared to determine the most appropriate temporal specification. For this model comparison, scale scores were standardized within each wave before equality constraints were imposed. M1 was an unconstrained model in which autoregressive and cross-lagged paths were freely estimated. M2 constrained autoregressive paths to be equal across adjacent intervals, M3 constrained cross-lagged paths to be equal across adjacent intervals, and M4 constrained both autoregressive and cross-lagged paths. The unconstrained model showed excellent fit and was retained because each constrained model showed a significant loss of fit relative to M1 and an increase in RMSEA greater than 0.015. Specifically, compared with M1, the likelihood-ratio tests were significant for M2, Δ*χ*^2^(3) = 16.58, *p* < 0.001, M3, Δ*χ*^2^(6) = 13.84, *p* = 0.031, and M4, Δ*χ*^2^(9) = 31.59, *p* < 0.001. Thus, the assumption that autoregressive and/or cross-lagged coefficients were invariant across the two adjacent intervals was not supported (Table 3).

**Table 3:** Model fit indices and model comparisons.

Model	chi-square	df	CFI	TLI	RMSEA	SRMR	Comparison	△ CFI	△ RMSEA
M1	6.94	6	1.000	0.998	0.017	0.007			
M2	23.51	9	0.994	0.980	0.055	0.015	M2-M1	−0.006	0.038
M3	20.77	12	0.997	0.991	0.037	0.017	M3-M1	−0.003	0.020
M4	38.53	15	0.991	0.980	0.054	0.027	M4-M1	−0.009	0.037

Model comparisons were conducted using scale scores standardized within each wave. M1 = unconstrained baseline model; M2 = model with autoregressive paths constrained to be equal across time; M3 = model with cross-lagged paths constrained to be equal across time; M4 = model with both autoregressive and cross-lagged paths constrained. CFI = Comparative Fit Index; TLI = Tucker–Lewis Index; RMSEA = Root Mean Square Error of Approximation; SRMR = Standardized Root Mean Square Residual.

Based on the retained M1 specification, we further estimated a covariate-adjusted CLPM in which gender and grade were included as time-invariant covariates. The covariate-adjusted model showed excellent fit, *χ*^2^(6) = 6.04, CFI = 1.000, TLI = 1.000, RMSEA = 0.003, and SRMR = 0.006. After controlling for gender and grade, the focal cross-lagged paths remained largely unchanged.

Fear of failure negatively predicted subsequent academic buoyancy from T1 to T2 (*β* = −0.247, *p* < 0.001) and from T2 to T3 (*β* = −0.237, p < 0.001). Academic buoyancy also negatively predicted subsequent fear of failure from T1 to T2 (*β* = −0.092, *p* = 0.011) and from T2 to T3 (*β* = −0.127, *p* = 0.001).

Academic buoyancy negatively predicted subsequent academic procrastination from T1 to T2 (*β* = −0.067, *p* = 0.048) and from T2 to T3 (*β* = −0.118, *p* = 0.008), whereas academic procrastination negatively predicted subsequent academic buoyancy from T1 to T2 (*β* = −0.232, p < 0.001) and from T2 to T3 (*β* = −0.105, *p* = 0.021).

The direct prospective paths between fear of failure and academic procrastination were weaker. Fear of failure positively predicted academic procrastination from T1 to T2 (*β* = 0.096, *p* = 0.012), but the T2-to-T3 path was non-significant (*β* = 0.033, *p* = 0.428). Academic procrastination positively predicted fear of failure from T1 to T2 (*β* = 0.201, *p* < 0.001), whereas the T2-to-T3 path was non-significant (*β* = 0.056, *p* = 0.144) (Figure 2).

**Figure 2:**
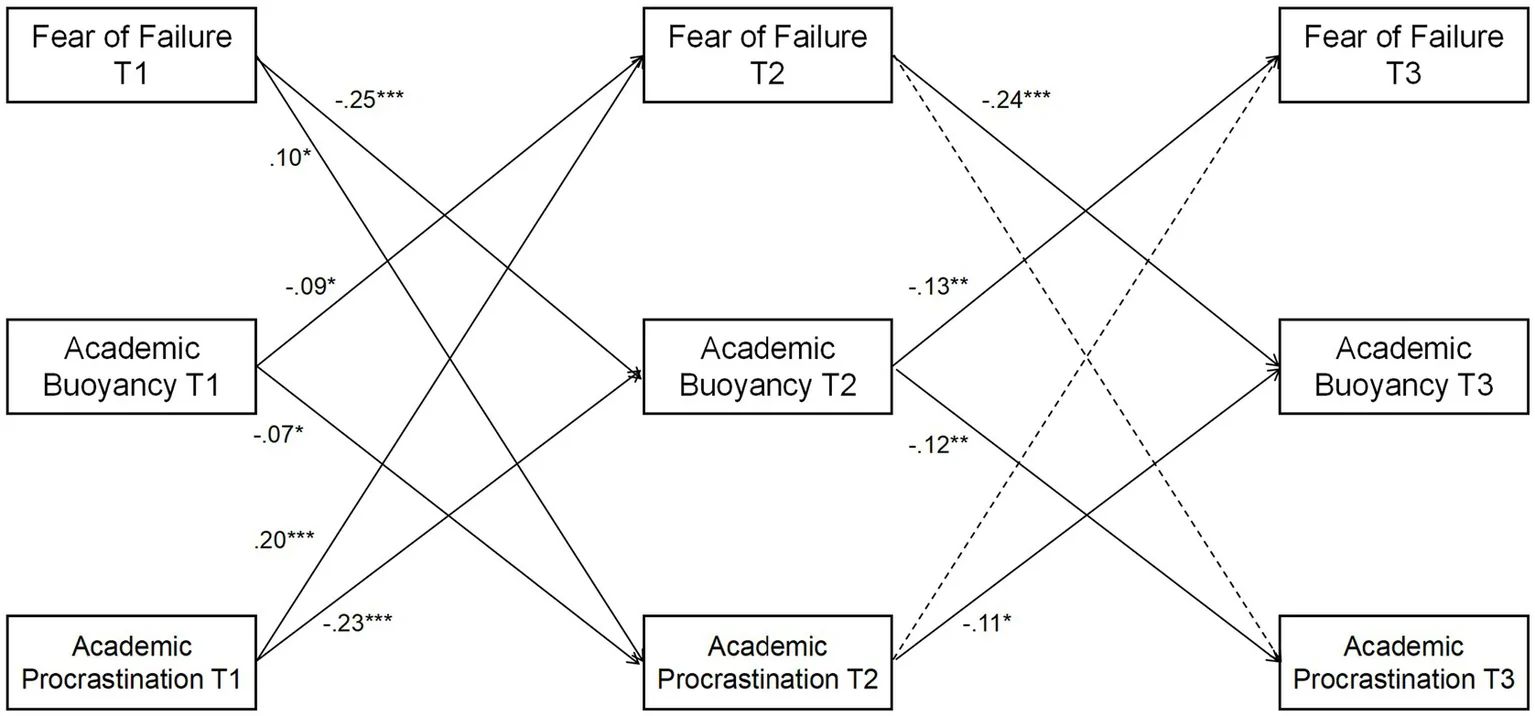
Covariate-adjusted cross-lagged associations among fear of failure, academic buoyancy, and academic procrastination. Values are standardized coefficients from the covariate-adjusted CLPM controlling for gender and grade. For readability, autoregressive paths, covariate paths, and within-wave correlations are not displayed. Dashed lines indicate non-significant paths; non-significant coefficients are omitted for clarity. *p < 0.05, **p < 0.01, ***p < 0.001.

### 3.7 Traditional CLPM-based longitudinal indirect associations

Traditional CLPM-based longitudinal indirect associations were evaluated using 5,000 bootstrap samples. In the covariate-adjusted CLPM, the indirect association from T1 fear of failure to T3 academic procrastination through T2 academic buoyancy was significant, *β* = 0.029, 95% bootstrap CI [0.005, 0.053]. The reverse indirect association from T1 academic procrastination to T3 fear of failure through T2 academic buoyancy was also significant, *β* = 0.030, 95% bootstrap CI [0.009, 0.050]. These results indicate significant longitudinal indirect associations through academic buoyancy in the traditional CLPM framework, even after accounting for gender and grade.

### 3.8 Supplementary robustness analysis

As a robustness analysis, we estimated a three-wave RI-CLPM to separate stable between-person differences from within-person fluctuations. The model fit the data well, *χ*^2^(3) = 0.28, CFI = 1.000, TLI = 1.011, RMSEA = 0.000, and SRMR = 0.002. However, the within-person cross-lagged effects were not uniform across the two adjacent intervals, and neither within-person longitudinal indirect effect was statistically significant. A summary of the RI-CLPM robustness analysis is reported in Supplementary Table S7.

## 4 Discussion

This three-wave longitudinal study examined reciprocal associations among fear of failure, academic buoyancy, and academic procrastination in upper elementary school students while accounting for autoregressive stability, gender, and grade. Overall, the findings support an integrated account in which fear of failure, academic buoyancy, and academic procrastination are prospectively linked over time. Academic buoyancy emerged as a central adaptive coping capacity that helped explain the longitudinal indirect associations between achievement-related threat appraisals and later procrastination in the traditional CLPM framework. The results also suggest that the pathways connecting fear of failure and procrastination may shift across the late-primary-school period.

Beyond the cross-lagged findings, exploratory analyses provided additional developmental context. Unconditional latent growth models indicated that, on average, fear of failure and academic procrastination increased across the three waves, whereas academic buoyancy decreased. Grade-level comparisons further showed that sixth-grade students reported higher fear of failure and academic procrastination and lower academic buoyancy than fifth-grade students. These descriptive patterns are consistent with increasing evaluative demands and academic pressure in late primary school, which may challenge students’ perceived capacity to cope with everyday academic setbacks. Because these analyses were descriptive, however, the primary temporal interpretations should be based on the cross-lagged models.

Common method bias diagnostics suggested that shared method variance was unlikely to explain the observed pattern of associations. Nevertheless, the use of self-report measures requires cautious interpretation, as discussed below.

### 4.1 Fear of failure and academic buoyancy: achievement threat and adaptive coping capacity

The study found reciprocal negative associations between fear of failure and academic buoyancy. This pattern is consistent with academic buoyancy theory and a COR-informed demands-resources perspective, suggesting that achievement-related threat appraisals and adaptive academic coping capacities may shape each other over time. Fear of failure may undermine later academic buoyancy because students who experience evaluation as threatening may allocate substantial cognitive and emotional effort to threat monitoring, rumination, and self-protection rather than to adaptive task coping (Sagar and Stoeber, 2009). When academic setbacks are interpreted as indicators of global inadequacy or social devaluation, students may be less able to recover quickly from ordinary academic difficulties.

Conversely, academic buoyancy predicted lower subsequent fear of failure. Buoyant students may be more likely to view ordinary academic setbacks as specific, temporary, and manageable rather than as evidence of personal inadequacy. This interpretation is consistent with work showing that academic buoyancy is linked to perceived control, persistence, and adaptive reappraisal (Martin et al., 2010; Martin et al., 2023). Thus, academic buoyancy should be understood not as a static psychological resource that is consumed by fear, but as a flexible coping capacity that can shape how students appraise and respond to everyday academic threats.

### 4.2 Academic buoyancy and academic procrastination: self-regulation under academic pressure

Academic buoyancy and academic procrastination also showed stable negative reciprocal associations. Higher buoyancy predicted lower later procrastination, suggesting that students who can manage ordinary academic setbacks may be less likely to rely on avoidance-based delay when tasks become difficult or emotionally aversive. Buoyant students may maintain engagement, use problem-focused coping, and preserve perceived control even when faced with pressure (Martin and Marsh, 2008; Nordby et al., 2017; Putwain et al., 2024).

The reverse pathway was also supported: higher procrastination predicted lower subsequent buoyancy. Procrastination may increase time pressure, incomplete task experiences, and perceived failure, thereby weakening students’ beliefs that they can manage ordinary academic challenges. In this way, procrastination may not only reflect self-regulatory difficulty but also contribute to later reductions in adaptive academic coping capacity.

### 4.3 Developmental considerations and shifts in pathways

Age and educational level are important contextual factors for interpreting the present findings. The current sample consisted of fifth- and sixth-grade students with a mean age of 10.74 years, representing a developmental period approaching the transition from primary to middle school. Much of the existing literature on academic procrastination has focused on university students, and evidence regarding its development at primary and secondary educational levels remains comparatively limited (Gonzalez-Brignardello et al., 2023). Similarly, although academic buoyancy is conceptually distinct from academic resilience, foundational research on academic buoyancy has largely been conducted with secondary-school students (Martin and Marsh, 2008). Thus, the meanings and dynamics of fear of failure, academic buoyancy, and procrastination in late primary school may differ from those observed in older adolescents or university students. During this period, increasing academic demands and expectations for independent learning may make students increasingly sensitive to evaluative pressure while placing greater demands on their capacity to cope with everyday academic setbacks. This developmental context may help explain the observed pattern in which fear of failure and procrastination increased across waves, academic buoyancy decreased, and sixth-grade students showed a less adaptive profile than fifth-grade students. However, because the present study was not designed to test age-related developmental change directly, these interpretations should remain cautious. Future longitudinal research spanning the transition from primary to middle school is needed to determine whether these reciprocal associations change systematically across age and educational levels.

A notable finding was that direct prospective associations between fear of failure and academic procrastination varied across waves. Fear of failure at T1 predicted procrastination at T2, whereas the direct T2-to-T3 path was not significant. One interpretation is that procrastination may become more stable over time. The autoregressive coefficient for procrastination from T2 to T3 was high (*β* = 0.70), indicating substantial temporal stability. As academic demands increase, procrastination may consolidate into a more habitual pattern, and behavioral inertia may reduce the direct contribution of fear-related emotions.

A second interpretation is that the pathway may increasingly involve adaptive coping capacity. During the period approaching the transition to middle school, fear of failure may shape procrastination less through a direct emotional route and more through its association with reduced academic buoyancy. This interpretation refines broad claims that fear of failure directly leads to procrastination by emphasizing the intervening role of academic coping capacity under sustained academic pressure (Haghbin et al., 2012; Zhao et al., 2023).

### 4.4 Academic buoyancy as a longitudinal indirect link

In the covariate-adjusted traditional CLPM, academic buoyancy showed significant longitudinal indirect associations linking fear of failure with later academic procrastination and linking academic procrastination with later fear of failure. This pattern is consistent with an integrated achievement motivation and demands-resources account: fear of failure may make academic demands more threatening, which may weaken students’ capacity to respond adaptively to everyday setbacks. Lower academic buoyancy may then impair goal-directed self-regulation and increase the likelihood of avoidance-based procrastination. The reverse indirect path suggests that procrastination may contribute to later fear of failure partly by undermining academic buoyancy. However, these indirect associations should be interpreted within the traditional CLPM framework rather than as definitive evidence of within-person causal mediation.

The supplementary RI-CLPM provided a more conservative test by separating stable between-person differences from within-person fluctuations. The RI-CLPM results indicated that some within-person cross-lagged paths remained significant, but the longitudinal indirect effects through academic buoyancy were not statistically significant. Thus, the traditional CLPM-based indirect-association findings should be understood as evidence of prospective indirect associations in the traditional CLPM, while the within-person indirect pathway requires further verification in future studies with more waves and larger samples.

### 4.5 Cultural considerations in a Confucian heritage context

The observed dynamics may also be shaped by sociocultural context. In Confucian Heritage Culture settings, academic achievement often carries strong social and familial meaning, including implications for family face, future mobility, and parental expectations (Ng et al., 2007; Hau and Ho, 2010). For students approaching the transition to middle school, academic failure may therefore be construed as particularly consequential. Such cultural meanings may intensify socially oriented fear of failure and make ordinary academic setbacks feel more threatening.

This cultural context may also shape academic buoyancy. Students who can maintain adaptive coping in the face of ordinary setbacks may be better positioned to manage not only task demands but also the social meanings attached to success and failure. Accordingly, explanations of procrastination among Chinese students should attend to both individual self-regulation and the broader cultural context in which failure is interpreted.

### 4.6 Educational implications

The findings have several implications for school-based interventions. First, interventions to reduce procrastination should not focus only on time management. Because academic buoyancy may help explain how fear of failure is linked to procrastination, school mental health and learning-support programs may benefit from strengthening students’ everyday academic coping capacity. Cognitive reappraisal training, attributional retraining, and problem-focused coping exercises may help students interpret routine setbacks as opportunities for adjustment rather than as global indicators of inadequacy (Putwain et al., 2012).

Second, interventions may need to be developmentally sensitive. For fifth-grade students, early work may focus on identifying and regulating fear of failure before avoidance becomes habitual. For sixth-grade students, interventions may additionally target perceived control, self-efficacy, and established patterns of delay. Teachers can support this process by setting attainable micro-goals, providing timely and specific feedback, and helping students experience progress in manageable steps (Martin, 2013; Martin et al., 2023).

Third, because demographic analyses suggested gender and grade-level differences in fear of failure and academic buoyancy, future intervention work may need to consider whether support should be differentiated across student groups. In particular, students reporting lower academic buoyancy or higher fear of failure may benefit from more targeted support for coping with evaluative pressure and ordinary academic setbacks (Salguero-Pazos and Reyes-de-Cozar, 2023).

### 4.7 Limitations and future directions

Several limitations should be considered when interpreting the findings. First, all variables were assessed through student self-report, which may be affected by social desirability, response bias, or shared method variance. Although common method bias diagnostics did not suggest a major problem, future studies should include multiple informants, such as parents and teachers, and behavioral indicators, such as homework submission timestamps, to enhance ecological validity.

Second, the study followed students only during late primary school. Although this period is developmentally important, longer longitudinal designs spanning the transition into middle school are needed to examine whether the associations among fear of failure, academic buoyancy, and procrastination change nonlinearly across development.

Third, although cross-lagged panel models provide stronger temporal evidence than cross-sectional designs, they remain correlational and cannot establish strict causal effects. Traditional CLPMs also do not fully separate stable between-person differences from within-person changes (Hamaker et al., 2015). Accordingly, the present findings should be interpreted as longitudinal predictive associations rather than evidence of causal effects. Although the supplementary RI-CLPM helped address this issue by separating stable between-person differences from within-person fluctuations, the model was based on only three waves and had limited degrees of freedom. Moreover, the within-person indirect effects were not statistically significant. Future studies with more measurement waves, shorter intervals, intervention designs, or quasi-experimental approaches are needed to clarify whether changes in academic buoyancy operate as a within-person mechanism linking fear of failure and academic procrastination.

Fourth, the sample was drawn from one primary school in Jiangxi Province, and the grade composition was uneven, with more fifth-grade than sixth-grade students. Although gender and grade were included as covariates in the primary CLPM, the single-school convenience sample limits the generalizability of the findings. Future studies should replicate the model in larger, more diverse, and more representative samples. Future work should also examine whether the structural paths are invariant across gender and grade groups using multi-group models or other sensitivity analyses.

## 5 Conclusion

This three-wave longitudinal study provides evidence of a prospective system linking fear of failure, academic buoyancy, and academic procrastination among upper elementary school students. After controlling for gender and grade, fear of failure and academic buoyancy showed negative reciprocal prospective associations, and academic buoyancy and academic procrastination were also negatively linked over time. In the traditional CLPM framework, academic buoyancy showed significant longitudinal indirect associations linking fear of failure with later procrastination and procrastination with later fear of failure. However, supplementary RI-CLPM results did not support statistically significant within-person indirect effects; therefore, the indirect associations should be interpreted cautiously. Overall, the findings highlight academic buoyancy as a potentially modifiable adaptive coping capacity that may help students manage achievement-related threat appraisals and reduce risk for maladaptive delay behavior in competitive educational contexts.

## Data Availability

The raw data supporting the conclusions of this article will be made available by the authors, without undue reservation.
