# Supplementary material for: Reciprocal associations among fear of failure, academic buoyancy, and academic procrastination in primary school students: a three-wave cross-lagged panel study
Source: Front Psychol. 2026 Oct 1;17:1847679. doi: 10.3389/fpsyg.2026.1847679 (PMC13669115; doi:10.3389/fpsyg.2026.1847679)
Supplement: Supplementary file 1 [file Data_Sheet_1.PDF]

Supplementary Materials

This document provides supplementary tables referenced in the main manuscript.

Table S1. Gender differences in study variables across waves.

Variable	Male M (SD)	Female M (SD)	t	p
T1 Fear of Failure	2.396 (1.113)	2.591 (1.032)	-2.109	.035
T2 Fear of Failure	2.387 (1.117)	2.704 (1.135)	-3.269	.001
T3 Fear of Failure	2.535 (1.095)	2.694 (1.187)	-1.618	.106
T1 Academic Buoyancy	3.708 (1.032)	3.813 (0.871)	-1.280	.201
T2 Academic Buoyancy	3.785 (0.957)	3.599 (0.997)	2.177	.030
T3 Academic Buoyancy	3.685 (1.054)	3.584 (0.996)	1.079	.281
T1 Academic Procrastination	1.995 (0.787)	1.962 (0.780)	0.488	.626
T2 Academic Procrastination	2.022 (0.808)	1.997 (0.807)	0.359	.720
T3 Academic Procrastination	2.069 (0.818)	2.136 (0.909)	-0.898	.370

Table S2. Grade differences in study variables across waves.

Variable	Grade 5 M (SD)	Grade 6 M (SD)	t	p
T1 Fear of Failure	2.402 (1.068)	2.781 (1.057)	-3.545	<.001
T2 Fear of Failure	2.426 (1.126)	2.925 (1.089)	-4.448	<.001
T3 Fear of Failure	2.559 (1.141)	2.792 (1.139)	-2.022	.044
T1 Academic Buoyancy	3.815 (0.928)	3.589 (1.020)	2.354	.019
T2 Academic Buoyancy	3.754 (0.974)	3.486 (0.978)	2.675	.008
T3 Academic Buoyancy	3.709 (1.017)	3.383 (1.019)	2.939	.003
T1 Academic Procrastination	1.921 (0.746)	2.159 (0.867)	-3.047	.002
T2 Academic Procrastination	1.937 (0.762)	2.236 (0.898)	-3.752	<.001
T3 Academic Procrastination	2.035 (0.846)	2.310 (0.891)	-3.204	.001

Table S3. Only-child status differences in study variables across waves.

Variable	Only child M (SD)	Non-only child M (SD)	t	p
T1 Fear of Failure	2.658 (0.898)	2.481 (1.089)	0.975	.330
T2 Fear of Failure	2.838 (1.192)	2.525 (1.130)	1.641	.101
T3 Fear of Failure	2.784 (1.015)	2.602 (1.153)	0.945	.345
T1 Academic Buoyancy	3.642 (0.969)	3.770 (0.954)	-0.785	.433
T2 Academic Buoyancy	3.678 (1.040)	3.692 (0.977)	-0.084	.933
T3 Academic Buoyancy	3.570 (1.012)	3.640 (1.028)	-0.368	.713
T1 Academic Procrastination	2.061 (0.792)	1.972 (0.782)	0.677	.498
T2 Academic Procrastination	2.209 (0.907)	1.994 (0.798)	1.586	.113
T3 Academic Procrastination	2.305 (0.918)	2.087 (0.860)	1.498	.135

Table S4. Unconditional latent growth model fit and parameter estimates.

Variable	chi-square	df	p	CFI	TLI	RMSEA	SRMR	Intercept mean	Slope mean	Intercept variance	Slope variance
Fear of Failure	.035	1	.852	1.000	1.004	.000	.001	2.494***	0.060**	.998***	.164***
Academic Buoyancy	.012	1	.914	1.000	1.007	.000	.001	3.760***	-0.072**	.520***	.087*
Academic Procrastination	2.428	1	.119	.999	.996	.051	.010	1.970***	0.060***	.508***	.037*

Note. Parameter estimates are reported with significance markers as in the source output (* p < .05, ** p < .01, *** p < .001).

Table S5. Common method bias diagnostics across waves.

Wave	Model	χ^2/df	TLI	CFI	RMSEA
T1	Without CLF	2.775	0.901	0.909	0.057
T1	With CLF	3.172	0.878	0.889	0.063
T2	Without CLF	2.841	0.918	0.925	0.058
T2	With CLF	3.426	0.892	0.902	0.067
T3	Without CLF	3.371	0.914	0.921	0.066
T3	With CLF	4.054	0.889	0.899	0.075

Note. CLF = common latent factor. Model fit indices are reported for the baseline model and the CLF model at each wave.

Table S6. Standardized cross-lagged paths from the covariate-adjusted CLPM.

Path	β	p
Fear of failure T1 → Academic buoyancy T2	-.247	<.001
Fear of failure T2 → Academic buoyancy T3	-.237	<.001
Academic buoyancy T1 → Fear of failure T2	-.092	.011
Academic buoyancy T2 → Fear of failure T3	-.127	.001
Academic buoyancy T1 → Academic procrastination T2	-.067	.048
Academic buoyancy T2 → Academic procrastination T3	-.118	.008
Academic procrastination T1 → Academic buoyancy T2	-.232	<.001
Academic procrastination T2 → Academic buoyancy T3	-.105	.021
Fear of failure T1 → Academic procrastination T2	.096	.012
Fear of failure T2 → Academic procrastination T3	.033	.428
Academic procrastination T1 → Fear of failure T2	.201	< .001
Academic procrastination T2 → Fear of failure T3	.056	.144

Note. The model controlled for gender and grade as time-invariant covariates predicting fear of failure, academic buoyancy, and academic procrastination across the three waves. β values are standardized coefficients from the covariate-adjusted cross-lagged panel model. CLPM = cross-lagged panel model; T1 = Time 1; T2 = Time 2; T3 = Time 3.

Table S7. RI-CLPM robustness analysis.

Outcome	Predictor	T1 -> T2 β	p	T2 -> T3 β	p
Fear of failure	Prior fear of failure	.341	.001	.386	<.001
Fear of failure	Academic buoyancy	-.009	.901	-.152	.026
Fear of failure	Academic procrastination	.301	.002	.057	.467
Academic buoyancy	Fear of failure	-.194	.044	-.282	.001
Academic buoyancy	Prior academic buoyancy	.040	.674	.139	.091
Academic buoyancy	Academic procrastination	-.293	.010	-.140	.089
Academic procrastination	Fear of failure	.161	.126	.160	.053
Academic procrastination	Academic buoyancy	-.118	.191	-.248	.002
Academic procrastination	Prior academic procrastination	.284	.047	.232	.022

Note. RI-CLPM = random-intercept cross-lagged panel model; CFI = Comparative Fit Index; TLI = Tucker-Lewis Index; RMSEA = Root Mean Square Error of Approximation; SRMR = Standardized Root Mean Square Residual. The RI-CLPM was estimated as a robustness analysis to separate stable between-person differences from within-person fluctuations. Neither within-person longitudinal indirect effect was statistically significant because the 95% bootstrap confidence intervals included zero.

Supplementary Note. As a robustness analysis, we estimated a three-wave RI-CLPM to separate stable between-person differences from within-person fluctuations. The model fit the data well, $\chi^2(3) = 0.28$, CFI = 1.000, TLI = 1.011, RMSEA = .000, and SRMR = .002. The within-person cross-lagged effects were not uniform across the two adjacent intervals. Moreover, neither within-person longitudinal indirect effect was statistically significant: fear of failure → academic buoyancy → academic procrastination, β = .048, 95% bootstrap CI [-.008, .104]; and academic procrastination → academic buoyancy → fear of failure, β = .044, 95% bootstrap CI [-.009, .098]. These findings suggest that part of the association identified in the traditional CLPM may reflect stable between-person differences and that within-person mediation should be interpreted cautiously.
